# Evolutionary conservation of Ebola virus proteins predicts important functions at residue level

**DOI:** 10.1093/bioinformatics/btw610

**Published:** 2016-09-21

**Authors:** Ahmed Arslan, Vera van Noort

**Affiliations:** KU Leuven, Center of Microbial and Plant Genetics, Leuven, Belgium

## Abstract

**Motivation:**

The recent outbreak of Ebola virus disease (EVD) resulted in a large number of human deaths. Due to this devastation, the Ebola virus has attracted renewed interest as model for virus evolution. Recent literature on Ebola virus (EBOV) has contributed substantially to our understanding of the underlying genetics and its scope with reference to the 2014 outbreak. But no study yet, has focused on the conservation patterns of EBOV proteins.

**Results:**

We analyzed the evolution of functional regions of EBOV and highlight the function of conserved residues in protein activities. We apply an array of computational tools to dissect the functions of EBOV proteins in detail: (i) protein sequence conservation, (ii) protein–protein interactome analysis, (iii) structural modeling and (iv) kinase prediction. Our results suggest the presence of novel post-translational modifications in EBOV proteins and their role in the modulation of protein functions and protein interactions. Moreover, on the basis of the presence of ATM recognition motifs in all EBOV proteins we postulate a role of DNA damage response pathways and ATM kinase in EVD. The ATM kinase is put forward, for further evaluation, as novel potential therapeutic target.

**Availability and Implementation:**

http://www.biw.kuleuven.be/CSB/EBOV-PTMs

**Supplementary information:**

Supplementary data are available at *Bioinformatics* online.

## 1 Introduction

Ebola Virus (EBOV) is a virus in the family of Filoviridae and causes severe hemorrhagic fever known as Ebola Virus Disease (EVD) with a mortality rate of up to 90%. EBOV is a rather small pathogen with only seven genes. The recent outbreak with epicenter in Guinea has sparked a number of new genomics studies (Gire *et al.*, 2014) that showed the virus proteins are not undergoing rapid evolution (Hoenen *et al.*, 2015) and mutations in different EBOV proteins are correlated with lethality (Deng *et al.*, 2015). These studies did not address highly conserved parts of proteins that we think may provide structural and functional insight into molecular functions of EBOV proteins.

There is no suitable medicine commercially available to date for EVD. Protein vaccines have failed to produce desired results in human subjects (Ponomarenko *et al.*, 2014). Recently, host calcium channels were shown to be involved in virus host-cell entry (Sakurai *et al.*, 2015) and were put forward as novel drug target. Host kinases also play a role in EVD, such as the PI3K kinase and PI3K/Akt pathways in EBOV proliferation (Saeed *et al.*, 2008). The interaction of ABL1 kinase with matrix protein VP40 has been linked to EBOV endocytosis (Garcia *et al.*, 2012). This association might facilitate morphogenesis and budding of new virions.

Different pathways and signaling cascades are routinely employed by viruses for infectivity (Lilley *et al.*, 2007). An example is the manipulation of Ras/MAPK pathways via IFN response that is associated with the elevation of EBOV infection in mice (Strong *et al.*, 2008). The activation of Mitogen associated protein kinase (MAPK) pathways like p38 pathway including DNA damage response are dependent on ATM (Munshi and Ramesh, 2013).

In order to gain more insight into the molecular biology of EVD we here aim to identify evolutionary conserved residues and assign functions to them. We do this by (i) collecting known and predicting novel post-translational modifications on EBOV proteins, (ii) mapping conserved residues on to three-dimensional structures, (iii) collect protein–protein interactions between virus and host proteins, (iv) find motifs that may mediate protein–protein interactions and (v) map conserved and modified residues to interaction interfaces.

## 2 Methods

We calculated residue level conservation for each Ebola protein based on 520 unique EBOV genomes. We annotated these with Post-translational Modifications and motifs and mapped them to known and modeled 3D-structures. For more details see Supplementary Methods.

## 3 Results

To identify important functions of individual residues in EBOV proteins, we combined residue conservation with several computational predictions and existing data.

### 3.1 Molecular functions of conserved residues

We created multiple sequences alignments for all EBOV proteins based on whole genome sequencing data of 520 EBOV genomes and identified highly conserved regions. Next, we annotated molecular functions to those conserved regions. Post-translational modifications (PTMs) are known for their role in protein activities and it has been shown that modified residues are more conserved than unmodified residues (Minguez *et al.*, 2012). We collected published data on modified residues in Ebola proteins. Only few PTMs have been characterized so far including thirteen phosphorylation sites; thus we extended the PTM repertoire by predicting novel posttranslational modifications. As EBOV proteins reside in the human host cell, we assume human modifying enzymes can modify them. We employed widely used neural network and SVM based published prediction methods trained on human PTM data to predict modified residues on EBOV proteins. We validated this strategy against published phosphorylation data; the prediction methods were able to predict eight out of thirteen published phosphorylation sites in EBOV (Supplementary Table S1). For further analyses we included modified residues in our final repertoire that had highest prediction value and all previously published PTMs.

**Fig. 1:**
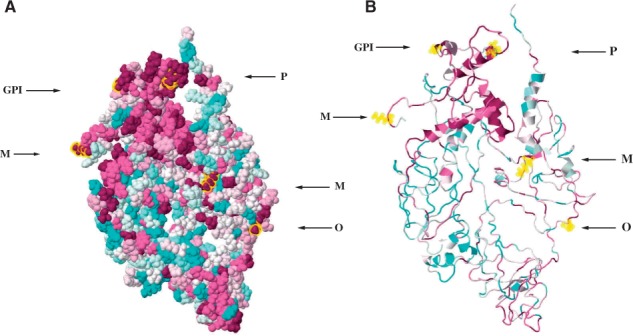
Ebola Glycoprotein (GP) conservation with post transitionally modified residues at conserved regions. PTMs present in regions of glycoprotein, conservation level indicated by dark blue, most variable residues; dark red, fully conserved sites; white, average conservation. Image created with ConSurf (Celniker *et al.*, 2013); (**A**) space filled and (**B**) backbone representation. (Abbreviation: P = phosphorylation, GPI = GPI-anchor, O = o-linked glycosylation, M = Methylation)

**Fig. 2:**
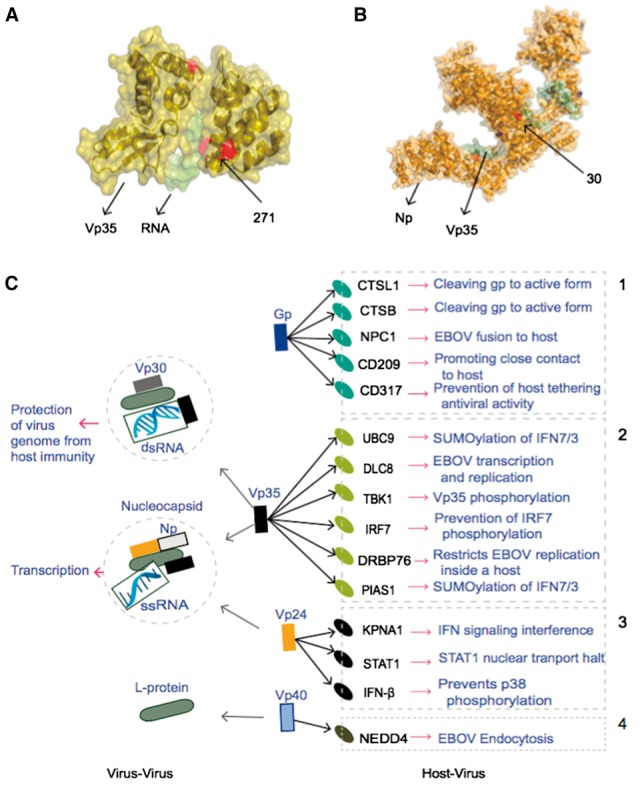
Ebola protein interactome. (**A**) The protein complex between VP35IID (brown) and dsRNA (green) (PDB*id*: 3L26); red residues are modified sites present in interface. (**B**) The protein complex between NP (brown)-VP35 (green) (PDB*id*: 4YPI), the PTM site present at interface shown in red. (**C**) The major interactions of EBOV proteins; left virus-virus, right host-virus. These interactions result in a number of different processes that either induce or suppress the host immune response. That is, some interactions are beneficial for the host whereas others are beneficial for the virus. The processes are grouped in boxes for each EBOV protein. Box1 shows the first step of attachment and entry of glycoprotein to host, boxes 2 and 3 are the interactive pathways to suppress the host immunity by vp35 and vp24, respectively, by interacting with host immune proteins like IRF7 (known for its role in interferon immunity); which may lead to the virus genome replication and transcription. 4, host NEDD4 play a possible role in virus budding and proliferation by interacting with and facilitating vp40 functions inside the host

The new list of PTMs is composed of 10 different types and 88 conserved PTM carrying amino acid residues in seven EBOV proteins (Fig. 1, Supplementary Table S2). Among predicted PTMs in EBOV proteins, phosphorylation is the most prevalent PTM type with 42 sites followed by methylation and o-glycosylation. Some of the PTMs are present in functional domains of EBOV proteins (Supplementary Table S2). Modified residues present at protein interaction interfaces may be important to virus proteins as immune escape is achieved through interaction with different host proteins (Fig. 2). The presence of different PTM-types on EBOV proteins like methylation and phosphorylation in glycoprotein (GP) can explain previously missing multiplicity of EBOV protein functions and regulation. Although previously, no specific role could be found for GP lipidation (Ito *et al.*, 2001), the conservation level suggests a crucial role, potentially in binding GP to lipid rafts together with the newly predicted conserved GPI-anchor site. The conserved SUMOylated residues in L-Protein could be responsible for nuclear localization for this protein.

Interestingly, for some of the modification types no sites were predicted at all, for example we did not predict any ubiquitination site for any of EBOV proteins. The simple explanation for that may be that virus proteins may avoid degradation and thus lack sequences that resemble ubiquitination signals.

### 3.2 Conserved residues in 3D structures

We map conservation from multiple sequence alignments of EBOV proteins to their protein tertiary structures (Celniker *et al.*, 2013). The tertiary structures of proteins were retrieved from PDB (Berman *et al.*, 2000). Only VP24 has a completely resolved protein structure whereas other EBOV proteins have partial structures either alone or in complex with other proteins in PDB (except for L protein). In order to evaluate the conservation of complete proteins in different structural domains, we extended our analyses to model complete EBOV proteins. We used a knowledge-based computational method to predict and refine the finished protein structures (Yang *et al.*, 2014; Zhang 2009; details see Supplementary Methods). EBOV proteins contain a variety of domains of which some are completely conserved and others are completely variable (Fig. 1, Supplementary Fig. S1). For example in Glycoprotein the unstructured regions have very low sequence conservation, whereas the helical parts are highly conserved. We annotate the known and predicted PTMs to conserved residues. This results in 14 modified residues present in known EBOV protein structures (Supplementary Table S3) and 88 if modeled structures are included (Supplementary Table S2).

### 3.3 Short motifs predict interactions with host proteins

Another approach to obtain mechanistic insight into the virus protein functions is based on finding short linear motifs. We employ Gibbs sampling (Davey *et al.*, 2010) and identify [ST]N.L.[FIV] known as Dok1 PTB domain binding motif in all EBOV proteins except for VP30 and twice in VP40. Dok proteins are adaptor molecules known for their role in regulation of signal transduction (Zhang *et al.*, 2004). Conserved PTB domains are found in many proteins and implicated for protein–protein interactions (Zhang *et al.*, 2004). Dok1 protein is known to bind through its PTB domain with protein kinases including Abelson tyrosine kinase (ABL1) (Cong *et al.*, 1999). ATM activates ABL1 (Shafman *et al.*, 1997) and ABL1 kinase phosphorylates many targets to facilitate cellular responses like intracellular mobility and host cell exit. Recently ABL1 was shown to phosphorylate the EBOV VP40 (Garcia *et al.*, 2012) needed for the proliferation of new virus particles during the EVD. The Dok1 PTB binding motif may be crucial for this phosphorylation.

A second motif NPG.C was only found in VP30, VP35 and L protein. This motif is called phosphotyrosine independent PTB domain motif and binds Dab-like PTB domains and functionally takes part in signaling pathways like endocytosis.

### 3.4 Modified residues at protein interaction interfaces

EBOV proteins interact with other virus proteins for spreading and with human proteins to evade host immunity or human proteins interact with EBOV proteins to accomplish immunity. To inspect the major interactions of EBOV proteins with host proteins or other virus proteins, we reconstructed the EBOV protein interactome based on literature review (Fig. 2, Supplementary Table S4). We hypothesize that the residues present at the interface should be more conserved and play a functional role in protein–protein interactions. We identified conserved residues with annotated PTMs in protein interaction interfaces of known virus-host and virus-virus protein complexes from PDB (Table 1). Three modified sites for glycoprotein are present in the interface with neutralizing antibody, one such site in the interface between NP-VP35, one modified residue at the interface of complex with VP40-Nedd4, one modified residue between VP24 and KPNA1 and one phosphosite in the same interface motif of two different 3D structures of the VP35 inhibitory domain with dsRNA (Supplementary Table S1). The presence of these predicted modifications at conserved residues of interface of a complex between two proteins suggest an important role of PTMs in EBOV protein complex formation.

**Table 1:** Post-translational modifications in protein interactions

Complex	PDB ID	PTM residues	Interface residue/motif
GP with antibody	3VE0	40	40
GP with antibody	3VE0	543	543
GP with antibody	3VE0	588	588
VP35 with Np	4PYI	30	30
VP40 with Nedd4	2KQ0	119	119
VP35 with dsRNA	4LG2	272	271 (motif)
VP35IID with dsRNA	4U2X	272	271 (motif)
VP24 with KPNA1	3L26	138	138

Modified residues overlap with residues in interaction interfaces in experimentally resolved 3D structures. Modified residue 272 of VP35 is next to the residue 271 that is part of an interaction interface.

### 3.5 ATMkinase central for EBOV functions

**Fig. 3:**
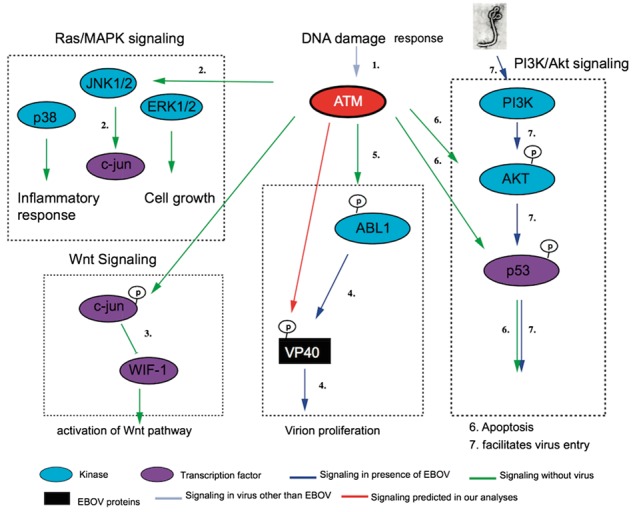
The role of ATM kinase in EVD related signaling pathways. Red arrows indicate novel interactions predicted in our analyses. Other arrows are derived from literature 1. (Xiaofei and Kowalik, 2014) 2. (Munshi and Ramesh, 2013) 3. (Svegliati *et al.*, 2014) 4. (Garcia *et al.*, 2012) 5. (Shafman *et al.*, 1997) 6. (Shiloh and Ziv, 2013) 7. (Saeed *et al.*, 2008)

We analyzed which kinases could be involved in the phosphorylation events of EBOV using published SVM based methods (Wong *et al.*, 2007). We identify three potential human kinases, including ATM (ataxia telangiectasia mutated), GSK3 (glycogen synthase 3 kinase) and CK2 (casein kinase 2). The ATM kinase could be linked to the modification of the majority of phosphorylation sites (Table 2). The ATM kinase is a major regulator of signaling pathways like DNA damage response (DDR) pathway (Supplementary Tables S6, S7) and interacts with other important kinases and transcription factors such as ABL1, ATR, AKT1, PI3K, p53 and MDM2 (van der Lee *et al.*, 2014; Shafman *et al.*, 1997; Shiloh and Ziv, 2013) (Fig. 3). ABL1 is a master regulator during the cytoplasmic mobility and ABL1 itself is phosphorylated by ATM kinase. Tyrosine phosphorylation of VP40 has been shown to be essential for Virion egress (Fig. 3, Garcia *et al.*, 2012). The p53 and MDM2 are integral part of apoptosis, cell cycle and PI3K/Akt signaling pathways. EBOV uses PI3K signaling for cellular entry (Saeed *et al.*, 2008). Finally, ATM has been shown to regulate wnt signaling (Svegliati *et al.*, 2014), both CK2 and GSK-3 are part of the wnt signaling pathway (Seldin *et al.*, 2005). Many viruses exploit DDR pathways for their own survival inside the host (reviewed elsewhere (Lilley *et al.*, 2007). Based on the presence of ATM Kinase motifs in EBOV proteins, we now suggest the activation of DDR pathways by EBOV as well (Fig. 3).

**Table 2:** Predicted host kinases and phosphorylated residues in EBOV proteins

Protein	ATM	CK2	GSK-3
**Gp**	59		449
**Vp24**	177	138	
**Vp30**	182, 184, 283		29
**Vp35**	272		
**Vp40**	119, 195, 316		
**NP**	193, 449, 679, 691	691	
**L**	750, 764, 913, 1036, 1264, 1303, 1993		

In this study we have identified highly conserved residues in EBOV proteins and explored their functional attributes by analysis of post-translational modifications, protein–protein interactions and linear motifs. The link with ATM Kinase motifs suggests the activation of DDR pathways in the presence of EBOV may trigger the possible ATM dependent phosphorylation cascades. Together these data suggest the potential of ATM as an interesting therapeutic target to be explored in the context of EVD.

## Funding

This work has been supported by the KU Leuven Research fund.

*Conflict of Interest*: none declared.

## Supplementary Material

Supplementary Data

## References

[btw610-B1] Berman H.M. et al (2000) The Protein Data Bank. Nucleic Acids Res., 28, 235–242. 10592235 10.1093/nar/28.1.235 PMC102472

[btw610-B2] Celniker G. et al (2013) ConSurf: using evolutionary data to raise testable hypotheses about protein function. Israel J. Chem., 53, p199–206.

[btw610-B3] Cong F. et al (1999) Characterization of a novel member of the DOK family that binds and modulates Abl signaling. Mol. Cell. Biol., 19, 8314–8325., p 10567556 10.1128/mcb.19.12.8314 PMC84915

[btw610-B4] Davey N.E. et al (2010) SLiMFinder: a web server to find novel, significantly over-represented, short protein motifs. Nucleic Acids Res., 38, W534–W539. 20497999 10.1093/nar/gkq440 PMC2896084

[btw610-B5] Deng L. et al (2015) Network of co-mutations in Ebola virus genome predicts the disease lethality. Cell Res., 1–4. 10.1038/cr.2015.54 PMC4456622 25976404

[btw610-B6] Garcia M. et al (2012) Productive replication of Ebola virus is regulated by the c-Abl1 tyrosine kinase. Sci. Transl. Med., 4, 123ra24–123ra24., p 10.1126/scitranslmed.3003500 PMC4794994 22378924

[btw610-B7] Gire S.K. et al (2014) Genomic surveillance elucidates Ebola virus origin and transmission during the 2014 outbreak. Science, 345, 1369–1372. 25214632 10.1126/science.1259657 PMC4431643

[btw610-B8] Hoenen T. et al (2015) Virology. Mutation rate and genotype variation of Ebola virus from Mali case sequences. Science, 348, 117–119. 25814067 10.1126/science.aaa5646 PMC11045032

[btw610-B9] Ito H. et al (2001) Ebola virus glycoprotein: proteolytic processing, acylation, cell tropism, and detection of neutralizing antibodies. J. Virol., 75, 1576–1580., p 11152533 10.1128/JVI.75.3.1576-1580.2001 PMC114066

[btw610-B10] van der Lee R. et al (2014) Classification of intrinsically disordered regions and proteins. Chem. Rev., 114, 6589–6631., p 24773235 10.1021/cr400525m PMC4095912

[btw610-B11] Lilley C.E. et al (2007) Using or abusing: viruses and the cellular DNA damage response. Trends Microbiol., 15, p119–126. 17275307 10.1016/j.tim.2007.01.003

[btw610-B12] Minguez P. et al (2012) Deciphering a global network of functionally associated post-translational modifications. Mol. Syst. Biol., 8, 599. 22806145 10.1038/msb.2012.31 PMC3421446

[btw610-B13] Munshi A., Ramesh R. (2013) Mitogen-activated protein kinases and their role in radiation response. Genes Cancer, 4, 401–408. p 24349638 10.1177/1947601913485414 PMC3863336

[btw610-B14] Ponomarenko J. et al (2014) Conservancy of mAb epitopes in Ebolavirus glycoproteins of previous and 2014 outbreaks. PLoS Currents, 6, 1–12. 10.1371/currents.outbreaks.f1a7028a13ce1c5f0bdbb4b0cc0b919b PMC4234462 25642381

[btw610-B15] Saeed M.F. et al (2008) Phosphoinositide-3 kinase-Akt pathway controls cellular entry of Ebola virus. PLoS Pathogens, 4, e1000141., 18769720 10.1371/journal.ppat.1000141 PMC2516934

[btw610-B16] Sakurai Y. et al (2015) Two-pore channels control Ebola virus host cell entry and are drug targets for disease treatment. Science, 347, 995–998., p 25722412 10.1126/science.1258758 PMC4550587

[btw610-B17] Seldin D.C. et al (2005) CK2 as a positive regulator of Wnt signalling and tumourigenesis. Mol. Cell Biochem., 274, 63–67., p 16342409 10.1007/s11010-005-3078-0

[btw610-B18] Shafman T. et al (1997) Interaction between ATM protein and c-Abl in response to DNA damage. Nature, 387, p520–523. 9168117 10.1038/387520a0

[btw610-B19] Shiloh Y., Ziv Y. (2013) The ATM protein kinase: regulating the cellular response to genotoxic stress, and more. Nat. Rev. Mol. Cell Biol., 14, 197–210. 23847781

[btw610-B20] Strong J.E. et al (2008) Stimulation of Ebola virus production from persistent infection through activation of the Ras/MAPK pathway. Proc. Natl. Acad. Sci. U. S. A., 105, 17982–17987., p 18981410 10.1073/pnas.0809698105 PMC2577702

[btw610-B21] Svegliati S. et al (2014) Oxidative DNA damage induces the ATM-mediated transcriptional suppression of the Wnt inhibitor WIF-1 in systemic sclerosis and fibrosis. Sci. Signal., 7, ra84. 25185156 10.1126/scisignal.2004592

[btw610-B22] Wong Y.H. et al (2007) KinasePhos 2.0: A web server for identifying protein kinase-specific phosphorylation sites based on sequences and coupling patterns. Nucleic Acids Res., 35. 10.1093/nar/gkm322 PMC1933228 17517770

[btw610-B23] Xiaofei E., Kowalik T.F. (2014) The DNA damage response induced by infection with human cytomegalovirus and other viruses. Viruses, 6, 2155–2185. p 24859341 10.3390/v6052155 PMC4036536

[btw610-B24] Yang J. et al (2014) The I-TASSER Suite: protein structure and function prediction. Nat. Methods, 12, 7–8. 10.1038/nmeth.3213 PMC4428668 25549265

[btw610-B25] Zhang Y. et al (2004) Molecular basis of distinct interactions between Dok1 PTB domain and tyrosine-phosphorylated EGF receptor. J. Mol. Biol., 343, 1147–1155., p 15476828 10.1016/j.jmb.2004.08.072

[btw610-B26] Zhang Y. (2009) Protein structure prediction: when is it useful? Curr. Opin. Struct. Biol., 19, 145–155. p 19327982 10.1016/j.sbi.2009.02.005 PMC2673339

