# Supplementary material for: Evolutionary conservation of Ebola virus proteins predicts important functions at residue level
Source: Bioinformatics. 2016 Sep 21;33(2):151–4. doi: 10.1093/bioinformatics/btw610 (PMC5942362; doi:10.1093/bioinformatics/btw610)
Supplement: Supplementary Data [file btw610_supp.zip › btw610-suppl_data/bioinformatics_Ebola_SI_v3.docx]

Evolutionary conservation of Ebola virus proteins predicts important functions at residue level

Ahmed Arslan and Vera van Noort

**Supplementary Methods**

*Ebola conservation analysis:* We collected genomic sequences of 545 Ebola genomes (Gire et al. 2014) of which 520 were unique. The gene DNA sequences of the 1976 strain were used to find homologs in these genome sequences with BLAST (Altschul et al. 1990) resulting in one homolog per genome for each of the Ebola genes. We made protein translations of all Ebola genes and homologs with EMBOSS tools (Goujon et al. 2010). We created multiple protein sequence alignments with ClustalOmega (McWilliam et al. 2013). From these alignments we calculated the evolutionary conservation for each position of the seven Ebola protein coding genes at the amino acid level compared to the reference sequence of 1976 (Celniker et al. 2013).

*Protein linear motif extraction study:* We extracted the short linear motifs in all the EBOV proteins using SlimFinder (Davey et al. 2010) software with default parameters. Extracted motifs were compared to all motifs present in the SlimFinder database with CompariMotif (Edwards et al. 2008) software.

*Post-Translational modifications data collection and prediction study:* To include all the previously published data on PTMs sites for Ebola virus proteins we performed a literature survey in PubMed (table S1)*.* In order to extend our repertoire of potential modified residues, we predicted the PTM sites with different servers, NetAcet for acetylation (Kiemer et al. 2005), NetCGlyc for c-mannosylation (Julenius 2007), NetGlycate for glycation (Johansen et al. 2006), big-PI Predictor for GPI-anchor (Eisenhaber et al. 1999), iMethyl-PreACC for methylation (W. R. Qiu et al. 2014), NetNGLyc for N-glycosylation, Yin-O-Yang for O-GLcNAc and NetOGlyc for O-glycosylation (Gupta and Brunak 2002), NBA-Palm for Palmitolyation (Xue et al. 2006), NetPhos and KinasePhos for phosphorylation and kinase specific prediction, respectively (Blom et al. 1999; Wong et al. 2007). Sulfinator for sulfation (Monigatti et al. 2002) PrePS for prenylation (Maurer-Stroh and Eisenhaber 2005), GPS-SUMO for sumoylation (Zhao et al. 2014) and UbPred for ubiquitination (Radivojac et al. 2010). These predictive methods are based on sequence based recognition searches of motifs specific for a particular PTM-type, combined with state of the art machine-learning methods either artificial neural network (Blom et al. 1999) or SVM based (Wong et al. 2007). We relied on the assumption that virus proteins undergo modifications inside the human cell and thus we specifically used human model based predictive methods. In our final list of PTMs, besides predicted modifications we also included all previously published PTM sites for EBOV proteins. To focus on the most reliably predicted PTMs, we included modified residues positions that are conserved in all the sequences, as previously shown PTMs carrying residues are more conserved (Minguez et al. 2012) and modified residues with SVM and neuronal network based predicted scores ranges from 0.5-1 (on the scale of 0-1), where 1 is the highest predictive value and 0.5 is the lowest). The reason to use such a stringent cut-off was to remove the possible noise in results and to focus on the most reliable modified residues.

*Protein structure data analysis:* The protein structure of VP24 was obtained from PDB (Berman et al. 2000) . For the other EBOV proteins we used protein modeling to obtain the protein structures. As a basis for modeling we used the protein sequences of the 1976 reference genome. We tried multiple homology modeling approaches but could not obtain good modeling results with these methods and thus relied on knowledge based *ab initio* structure prediction using the I-TASSER suite 4.3 (Yang et al. 2014) from the Zhang lab. I- TASSER uses the experimentally resolved 3D-structure information from proteins in PDB with similar folds, in case of missing folds it uses *ab initio* modeling. That is, the structural templates from experimental data for a provided amino acid sequence and (where the experimental data was missing it performed) *ab initio* modeling and templates were stitched together on the bases of intermolecular energy factor and simulated by Monte Carlos simulation (Yang et al. 2014). The final solutions for proteins structure predictions included all the available data for proteins structures present in PDB including the secondary structure information present (supplementary file1-Note1). Non-globular structures like intrinsically disordered regions are also included where the information is available in PDB (supplementary file1-Note1). The server ranks the prediction solutions according to their TM align-score, a TM-score of two structures higher than 0.5 represent proteins with the same folds. The structures of proteins with highest prediction confidence scores were selected for downstream analyses (table S8). High TM-scores were obtained for GP, L and NP proteins (TM > 0.7), good TM-scores for Vp30 and VP40 (TM>0.5) and a reasonable score for Vp35 (TM=0.42). Furthermore, we validated each structure with validation algorithms using QMEAN (Benkert et al. 2008). Conservation was assigned to each amino acid for all the protein structures using the ConSurf program (fig. 1, fig. S1) (Celniker et al. 2013).

*Protein docking analysis:* Where available experimental data for EBOV protein complexes was retrieved from PDB. Where the data was lacking or only partially available, protein-protein docking was performed by GRAMM-X, an improved version of Global RAnge Molecular Matching (GRAMM) (Tovchigrechko and Vakser 2006; Bhattacharya et al. 2010). GRAMM is well known efficient and easy to use platform for protein complex docking; it uses the energy function as primary basis for protein-protein complex assembly but the final docking solution also includes the evolutionary conservation of interacting residues and statistical residue-residue preference besides lowest energy calculations. We used the server with default parameters; the GRAMM-X docking results in multiple modeling solutions that are ranked based on the average sum of the Lennard-Jones term, evolutionary conservation and statistical residue-residue preference. We performed further analyses and data interpretation with the top-ranked models “MODEL 1” from output in each case. (For proteins structures (and complexes) files in pdb format see supplementary information). The visualization of alignments, protein structure and interaction surfaces of protein complexes were performed in Jalview (Waterhouse et al. 2009), Jmol (Hanson 2010) and PyMol (DeLano 2014), respectively.

**Supplementary Discussion**

In this study we have identified the most conserved residues in EBOV proteins and explored their functional attributes by analysis of post-translational modifications, protein-protein interactions and linear motifs. We related these functional residues to the exploitation of host resources during infection, like modifying enzymes (kinases) and pathways. We predict novel modified residues, identify short interaction motifs and model complete protein structures and interacting proteins.

In some areas substantial data is missing for EBOV proteins such as complete protein three-dimensional structures; this leads to an underestimation in our analyses of residues involved in protein interactions. Nevertheless, the results presented here expand our present understanding of functional annotation of individual residues for modifications and protein interactions.

The most frequent kinase that appeared in our analysis of predicted kinases responsible for virus protein modification was the double stranded damage response elements (ATM), whether the interaction of ATM with ABL1 during DNA damage response (Shafman et al. 1997) or PI3K signaling (Saeed et al. 2008) or Ras/MAPK pathways (Munshi and Ramesh 2013), ATM emerges as a central regulator of EVD through these different but closely related pathways (fig. 3). Other kinases that potentially phosphorylate the EBOV proteins, GSK3 and CK2, are part of Wnt signaling pathway (Seldin et al. 2005). Previously, GSK3 kinase has been implicated in antiviral activity including EVD (Sun et al. 2012). On one side, it is reported that the Wnt signaling is crucial for viral infection (Angelova et al. 2012) and on the other hand, the ATM is also shown to be associated with Wnt signaling activation via inhibition of the WIF1, an inhibitor of Wnt signaling, in human cancer cells (Svegliati et al. 2014).

The presence of ATM recognition motifs in all EBOV proteins suggests an intersection of these closely associated pathways with DDR pathways. Many viruses exploit DDR pathways for their own survival inside the host (reviewed elsewhere (Lilley et al. 2007). Briefly, during viral invasion, ATM present in the host cell is activated upon entry of “alien” genomic material, which is signaled to ATM as DNA damage. Both, DNA and RNA viruses modulate the host ATM-dependent DDR activity (Xiaofei and Kowalik 2014). This modulation serves two purposes for viral survival; firstly, it extends the S-phase of an invaded cell by regulating the host cell cycle checkpoints which ensures viral replication (Dahl et al. 2005) and secondly, it stops infected cell to enter apoptosis until required, by preventing the phosphorylation of pro-apoptosis proteins, p53 and p38 (Lee et al. 2015). VP24 also prevents phosphorylation of p38 by blocking IFN signaling (Halfmann et al. 2011) and our analysis shows the VP24 itself phosphorylated by ATM kinase (table 3). Based on these data, we suggest the activation of DDR pathways in the presence of EBOV may trigger the possible ATM dependent phosphorylation cascades involving activations of many pathways and proteins to help virus proliferation (fig S3). Together these data suggest the potential of ATM as an interesting therapeutic target to explore its curative potency for EVD.

Signaling is a complex affair and involves multiple overlapping pathways, and we propose ATM as a “junction” signaling kinase (concept introduced by Nishi et al (Nishi et al. 2015)) between several pathways in EVD. That is, during EVD, the activation of DDR (and possibly of Wnt signaling pathways as well) via ATM kinase and downstream processing. Based on our results and current understanding of pathways an association is anticipated of ATM kinase with pathways and kinases through which Ebola pathology is achieved (Saeed et al. 2008; s et al. 2012). Cellular Ebola virus infection assays in the context of ATM knockout or knockdown cell-lines could validate this model in the future.

**Table S1:** Overlap between predicted PTMs and previously reported phosphorylation and other modified sites. The second column shows all sites that have been predicted with score >0.5.

| **Protein** | **Experimental phospho-site** | **Prediction** | **Predictive value** | **Relative conservation (ConSurf)** | **ConSurf color gradient** | **Reference** |
| --- | --- | --- | --- | --- | --- | --- |
| **Vp30** | S29 | Yes | 0.95 | -0.610 | 9 | Ilinykh, 2014 |
|  | S30 | Yes | 0.997 | -0.370 | 3 | Ilinykh, 2014 |
|  | S31 | Yes | 0.997 | -0.478 | 8 | Ilinykh, 2014 |
|  | S42 | Yes | 0.785 | 0.665 | 1 | Ilinykh, 2014 |
|  | S46 | Yes | 0.970 | -0.516 | 8 | Ilinykh, 2014 |
|  | T52 | Yes | 0.595 | -0.639 | 9 | Ilinykh, 2014 |
| **Np** | T563 | Yes | 0.952 | -0.437 | 8 | Peyrol et al., 2013 |
|  | S647 | Yes | 0.997 | -0.013 | 5 | Peyrol et al., 2013 |
| **Gp** | 40 | Yes | 0.74 | -1.328 | 9 | Jeffers et al. 2002 |
|  | 204 | Yes | 0.68 | -0.647 | 7 | Jeffers et al. 2002 |
|  | 228 | Yes | 0.60 | 1.351 | 1 | Jeffers et al. 2002 |
|  | 238 | Yes | 0.71 | -0.844 | 8 | Jeffers et al. 2002 |
|  | 257 | Yes | 0.71 | -0.858 | 8 | (Jeffers et al. 2002)(Jeffers et al. 2002)(Jeffers et al. 2002)(Jeffers et al. 2002) Jeffers et al. 2002 |
|  | 268 | Yes | 0.61 | -0.730 | 7 | Jeffers et al. 2002 |
|  | 317 | Yes | 0.66 | 1.419 | 1 | Jeffers et al. 2002 |
|  | 386 | Yes | 0.67 | 1.573 | 1 | Jeffers et al. 2002 |
|  | 436 | Yes | 0.60 | 1.288 | 1 | Jeffers et al. 2002 |
|  | 454 | Yes | 0.50 | -0.876 | 8 | Jeffers et al. 2002 |
|  | 563 | Yes | 0.52 | -1.328 | 9 | Jeffers et al. 2002 |
|  | 672 | Yes | 0.799 | -0.936 | 8 | Ito et al. 2001 |

**

**

**Fig. S1: The conservation of EBOV proteins.** The conservation calculated with ConSurf (Celniker et al. 2013) protocol shown in color gradient, from dark red to dark blue, as most conserved to most variable respectively.

**Table S2: The conserved PTM-types predicted in different EBOV proteins.** Predicted PTMs present in different EBOV protein including their functional regions.

**
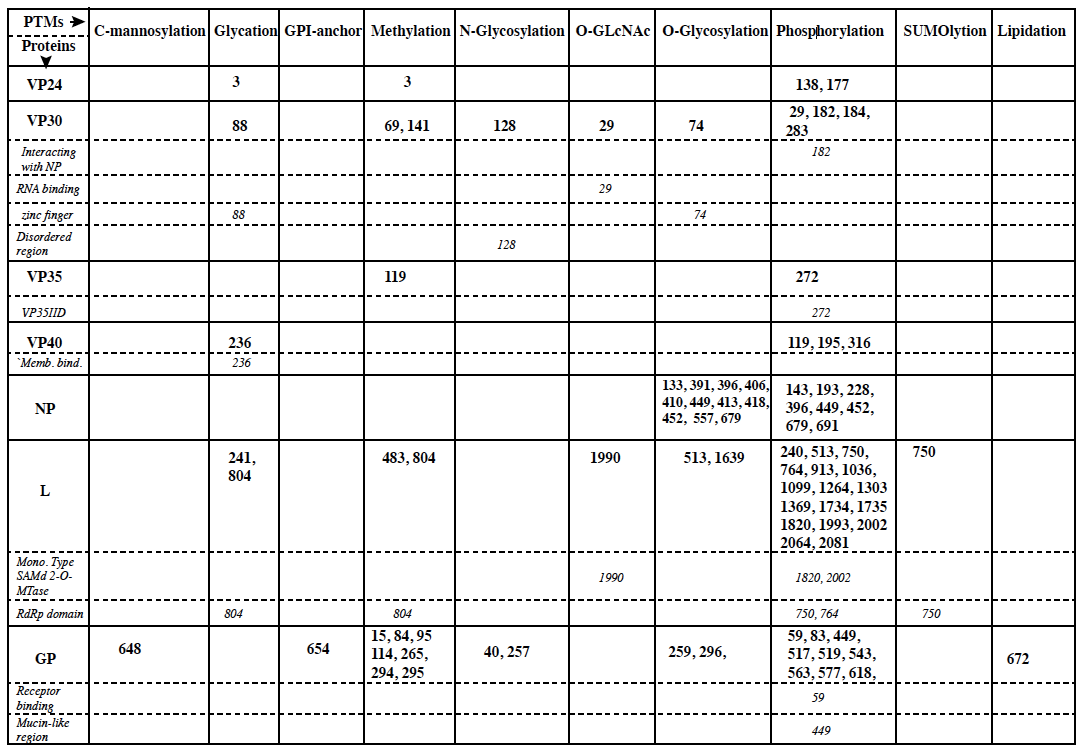
**

**Table S3: Predicted phosphorylation sites in EBOV protein structures available in PDB.**

| **Protein** | **PDB ID** | **Sequence** | **PTM positions** | **Reference of PDB structure** |
| --- | --- | --- | --- | --- |
| **Vp30** | 2I8B | 120-272 | 182, 184 | Hartlieb et al 2007 |
| **Gp** | 3CSY | 501-632 | 517, 519, 543, 563, 577, 618 | Lee J E 2008 |
| **Np** | 4QB0 | 638-739 | 679, 691 | Dziubanska 2014 |
| **Vp35** | 3FKE | 211-340 | 272 | Leung DW 2009 |
| **Vp24** | 3VNE | 9-232 | 138, 177 | Zhang 2012 |
| **Vp40** | 4LDD | 30-327 | 195 | Bornholdt 2013 |

**Table S4**: **Virus-host protein-protein interactions** based on literature mining*(v = virus, h = host)

| **h-protein*** | **v-protein** | **Functions of h-protein** | **Consequences of interaction** | **Reference** |
| --- | --- | --- | --- | --- |
| DLC8 | VP35 | Intracellular Viral transport/replication | EBOV transcription/replication machinery | (Kubota et al. 2009; Luthra et al. 2013) |
| dsRNA | VP35 | Viral replication/transcription | Hide viral genome from host- cellular sensors | (Bale et al. 2013) |
| UBC9 | VP35 | SUMO E2 | SUMOylation of IRF7/3 upon interaction with EROV | (Chang et al. 2009) |
| TBK1 | VP35 | Phosphorylation of IRF7/3 | Phosphorylates EBOV vp35 | (Prins et al. 2009) |
| IRF7 | VP35 | Immune response | Prevents IRF7 phosphorylation and activation of cellular response | (Leung et al. 2011) |
| DRBP76 | VP35 | Inhibits the viral replication | Binds and restrict EBOV replication | (Shabman, et al. 2011) |
| PIAS1 | VP35 | SUMO E3 | SUMOylation of IRF7/3 upon interaction with EROV | (Chang et al. 2009) |
| KPNA1 | VP24 | Protein transportation to nucleus | Prevents transportation of STAT1 | (Shabman, et al. 2011) |
| STAT1 | VP24 | Host cellular immunity | Disturbance in IFN signaling | (Zhang et al. 2012) |
| CTSL1 | GP | Protein catabolism | Cleaving full length EBOV gp | (Chandran et al. 2005) |
| CTSB | GP | Protein catabolism | Cleaving full length EBOV gp | (Chandran et al. 2005) |
| NPC1 | GP | Transportation | EBOV membrane fusion to host | (Carette et al. 2011) |
| CD209 | GP | Cell surface receptor | Promoting close contact of EBOV with host cell | (Marzi et al. 2007) |
| CD317 | GP | Anti-viral host restriction | Prevention of tethering activity | (Gnirß et al. 2014) |
| Nedd4 | VP40 | Ubiquitination | Endocytosis | (Harty et al. 2000) |

**Table S5:** **PTMs at conserved sites from protein docking data of EBOV-Host interactome** (v = virus, *h* = host) The h-v shows the modified residues from both host and virus present at interface. *same residue of 272.

| **v-proteins** | **Vp35** | **Vp40** | **Vp30** | **dsRNA** | *h* ***-* v** | ***h*-proteins** |
| --- | --- | --- | --- | --- | --- | --- |
| Vp35 | - | - | - | -- | *88, 64, 36, 43, 547, 777, 804* – **0** | *DLC8* |
| Vp35 | - | - | - | **0**- 271* | *344* – **272** | *TBK1* |
| Vp35 | - | - | - |  | *67* – **0** | *DRBP76* |
| Vp35 | - | - | **-** | **-** | *0* – **272** | *IRF7* |
| Vp35 | - | - | **-** | **-** | *0* – **272** | *UBC9* |
| L | **272 -** 804 | - | **88, 128, 184** – 0 | - | - | *PIAS1* |
| NP | **-** | - | **182, 184**- 0 | - | - | *-* |
| Vp40 |  |  |  |  | 0-**119** | *NEDD4* |
| Vp24 | - | - | - | - | *396, 398* – **0** | *KPNA1* |
| Gp | - | - | - | - | *106* – **40** | *CD317* |

**Table S6: The ATM interactome.** Enrichment analysis of ATM and interacting partners of ATM in different pathways with false discovery rate (FDR)

| **Pathways** | **Proteins** | **FDR** |
| --- | --- | --- |
| FC receptor signaling pathway | ABL1, PI3K, AKT1, MDM2 | 2.3e-4 |
| Signal transduction in response to DNA damage | ATM, ABL1, MDM2, P53 | 1.6e-3 |
| PI3K activity | ATM, PI3K | 1.6e-3 |
| Cell phase transition | ATM, AKT1, MDM2, p53 | 7.05e-3 |
| Cell cycle arrest | ATM, p35, MDM2 | 8.94e-3 |
| Wnt signaling | GSK3B, CSNK2A1 | 1.22e-5 |

**Table S7: Interactions of ATM with individual kinases** (different colors of kinases show different pathways)

| **Pathways** | **Protein in complex with ATM** | **FDR** |
| --- | --- | --- |
| Signaling in response to DNA damage | ABL1 | 2.65e-4 |
| PI3-Kinase activity | PI3K | 2.49e-4 |
| Cell check points | Akt1 | 2.3e-7 |
| Regulation of cell response in stress | ERK1 | 3.03e-7 |
| Signaling in DNA damage response | p38 | 1.20e-3 |
| Cell check points | CSNK2A1 | 2.2e-5 |

*DNA damage response/PI3K/Atk pathway; Ras/MAPK pathway, Wnt signaling





**Fig S2: The ATM Interactome.** The protein interactions shown for different kinases including ATM, PI3K and ABL1 (Mostafavi et al. 2008).

**Table S8: Quality control of protein structure predictions by I-TASSER.** The PDB ID col. shows the known structures models of EBOV present in PDB. The c-score is a confidence score for protein structure prediction approximation by the I-TASSER algorithm (see Materials and Methods) and ranges from -5 to 2; a higher value signifies a structure prediction with higher confidence and vice-versa. TM-score is a standard scale for measuring similarities between two structures (predicted and native), a TM-score >0.5 has correct topology and TM<0.17 represents random similarity. *(Vp24 model was taken from PDB (ID: 3VNE))

| **Proteins** | **PDB ID** | **C-score** | **TM-score** |
| --- | --- | --- | --- |
| **Gp** | 3CSY | 0.14 | 0.73 |
| **L-Protein** | Not available | -0.13 | 0.70 |
| **Np** | 4QB0 | 0.19 | 0.74 |
| **Vp24*** | 3VNE | Used from PDB | Used from PDB |
| **Vp30** | 2I8B | -1.75 | 0.8 |
| **Vp35** | 3FKE | -2.51 | 0.42 |
| **Vp40** | 4LDD | -0.39 | 0.66 |

**Note 1: Protein modeling and experimental basis**

The protein models data were built on top of experimentally resolved protein models available in PDB. The first step of I-TASSER protocol is to search for available structural templates (J Yang et al Nature 2015). If a template is found in PDB, I-TASSER includes it in the final model (see materials and methods). The protein models generated in our analyses include available experimental models. For example, the following VP35 structure (in red) is the one we predicted through protein modeling and the blue structure is the protein model of VP35IID that is available in PBD (ID: 3FKE).





The scores (TM score) for the alignment between predicted and experimentally resolved protein structures present in PDB are listed in table S9.

We also included the structural information regarding disordered regions only if it is experimentally resolved and available in PDB. For example, the protein complex between NP and VP35 (PDB ID: 4YPI), contained a 28 amino acid long intrinsically disordered region of Vp35 from position 20 to 48 (in blue), the overlap between this region and the predicted VP35 protein structure yielded a TM-align score of 0.38 that represents a score in-between a random overlap and in the same fold.

A

 B
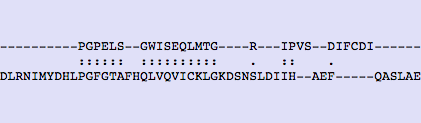


(A) The overlap of the regions of predicted VP35 structure (red) and VP35 chains from 4YPI model (blue). (B) The overlap of 28 amino acid long sequence of VP35 chain from the 4YPI model (upper) with the predictive model of VP35 (lower).

VP30 has few regions of intrinsically disorder and our analysis on the VP30 structure prediction includes the information regarding these regions where the information in present in PDB. The VP30 experimentally resolved structure (2I8B) covers the regions from 120 to 270 that explicitly are disordered regions and our structural alignments yield a high score of overlap between these two structural templates (0.82)

A



B
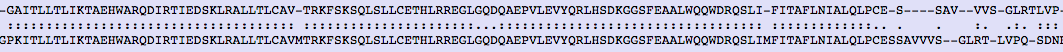


(A) The blue template represents the PDB structure of VP30 (2I8B) and red one represents our structure solution for VP30. This score is already included in the table S9 (B) The alignment of two sequences, the upper sequence is of VP30 (2I8B) and the lower one is the region from predicted VP30 model.

**References:**

Altschul SF, Gish W, Miller W, Myers EW, Lipman DJ. 1990. Basic local alignment search tool. J. Mol. Biol. [Internet] 215:403–410.

Angelova M, Zwezdaryk K, Ferris M, Shan B, Morris C a, Sullivan DE. 2012. Human cytomegalovirus infection dysregulates the canonical Wnt/β-catenin signaling pathway. PLoS Pathog. 8:e1002959.

Bale S, Julien J-P, Bornholdt Z a, Krois AS, Wilson I a, Saphire EO. 2013. Ebolavirus VP35 coats the backbone of double-stranded RNA for interferon antagonism. J. Virol. 87:10385–10388.

Benkert P, Tosatto SCE, Schomburg D. 2008. QMEAN: A comprehensive scoring function for model quality assessment. Proteins 71:261–277.

Berman HM, Westbrook J, Feng Z, Gilliland G, Bhat TN, Weissig H, Shindyalov IN, Bourne PE. 2000. The Protein Data Bank. Nucleic Acids Res. 28:235–242.

Bhattacharya M, Biswas A, Das AK. 2010. Interaction analysis of TcrX/Y two component system from Mycobacterium tuberculosis. Biochimie 92:263–272.

Blom N, Gammeltoft S, Brunak S. 1999. Sequence and structure-based prediction of eukaryotic protein phosphorylation sites. J. Mol. Biol. 294:1351–1362.

Carette JE, Raaben M, Wong AC, Herbert AS, Obernosterer G, Mulherkar N, Kuehne AI, Kranzusch PJ, Griffin AM, Ruthel G, et al. 2011. Ebola virus entry requires the cholesterol transporter Niemann-Pick C1. Nature 477:340–343.

Celniker G, Nimrod G, Ashkenazy H, Glaser F, Martz E, Mayrose I, Pupko T, Ben-Tal N. 2013. ConSurf: Using evolutionary data to raise testable hypotheses about protein function. Isr. J. Chem. 53:199–206.

Chandran K, Sullivan NJ, Felbor U, Whelan SP, Cunningham JM. 2005. Endosomal proteolysis of the Ebola virus glycoprotein is necessary for infection. Science 308:1643–1645.

Chang TH, Kubota T, Matsuoka M, Jones S, Bradfute SB, Bray M, Ozato K. 2009. Ebola Zaire virus blocks type I interferon production by exploiting the host SUMO modification machinery. PLoS Pathog. 5.

Dahl J, You J, Benjamin TL. 2005. Induction and utilization of an ATM signaling pathway by polyomavirus. J. Virol. 79:13007–13017.

Davey NE, Haslam NJ, Shields DC, Edwards RJ. 2010. SLiMFinder: a web server to find novel, significantly over-represented, short protein motifs. Nucleic Acids Res. 38:W534–W539.

DeLano WL. 2014. The PyMOL Molecular Graphics System.

Edwards RJ, Davey NE, Shields DC. 2008. CompariMotif: Quick and easy comparisons of sequence motifs. Bioinformatics 24:1307–1309.

Eisenhaber B, Bork P, Eisenhaber F. 1999. Prediction of potential GPI-modification sites in proprotein sequences. J. Mol. Biol. 292:741–758.

Garcia M, Cooper a., Shi W, Bornmann W, Carrion R, Kalman D, Nabel GJ. 2012. Productive Replication of Ebola Virus Is Regulated by the c-Abl1 Tyrosine Kinase. Sci. Transl. Med. 4:123ra24–ra123ra24.

Gire SK, Goba A, Andersen KG, Sealfon RSG, Park DJ, Kanneh L, Jalloh S, Momoh M, Fullah M, Dudas G, et al. 2014. Genomic surveillance elucidates Ebola virus origin and transmission during the 2014 outbreak. Sci. 345 :1369–1372.

Gnirß K, Fiedler M, Krämer-Kühl A, Bolduan S, Mittler E, Becker S, Schindler M, Pöhlmann S. 2014. Analysis of determinants in filovirus glycoproteins required for tetherin antagonism. Viruses 6:1654–1671.

Goujon M, McWilliam H, Li W, Valentin F, Squizzato S, Paern J, Lopez R. 2010. A new bioinformatics analysis tools framework at EMBL-EBI. Nucleic Acids Res. 38.

Gupta R, Brunak S. 2002. Prediction of glycosylation across the human proteome and the correlation to protein function. Pac. Symp. Biocomput. 322:310–322.

Halfmann P, Neumann G, Kawaoka Y. 2011. The Ebolavirus VP24 protein blocks phosphorylation of p38 mitogen-activated protein kinase. J. Infect. Dis. 204:953–956.

Hanson RM. 2010. Jmol-a paradigm shift in crystallographic visualization. J. Appl. Crystallogr. 43:1250–1260.

Harty RN, Brown ME, Wang G, Huibregtse J, Hayes FP. 2000. A PPxY motif within the VP40 protein of Ebola virus interacts physically and functionally with a ubiquitin ligase: implications for filovirus budding. Proc. Natl. Acad. Sci. U. S. A. 97:13871–13876.

Ito H, Watanabe S, Takada A, Kawaoka Y. 2001. Ebola virus glycoprotein: proteolytic processing, acylation, cell tropism, and detection of neutralizing antibodies. J. Virol. 75:1576–1580.

Jeffers S a, Sanders DA, Sanchez A. 2002. Covalent modifications of the ebola virus glycoprotein. J. Virol. 76:12463–12472.

Johansen MB, Kiemer L, Brunak S. 2006. Analysis and prediction of mammalian protein glycation. Glycobiology 16:844–853.

Julenius K. 2007. NetCGlyc 1.0: Prediction of mammalian C-mannosylation sites. Glycobiology 17:868–876.

Kiemer L, Bendtsen JD, Blom N. 2005. NetAcet: Prediction of N-terminal acetylation sites. Bioinformatics 21:1269–1270.

Kubota T, Matsuoka M, Chang T-H, Bray M, Jones S, Tashiro M, Kato A, Ozato K. 2009. Ebolavirus VP35 interacts with the cytoplasmic dynein light chain 8. J. Virol. 83:6952–6956.

Lee R Van Der, Buljan M, Lang B, Weatheritt RJ, Daughdrill GW, Dunker a K, Fuxreiter M, Gough J, Gsponer J, Jones DT, et al. 2015. Classi fi cation of Intrinsically Disordered Regions and Proteins.

Leung LW, Park M-S, Martinez O, Valmas C, López CB, Basler CF. 2011. Ebolavirus VP35 suppresses IFN production from conventional but not plasmacytoid dendritic cells. Immunol. Cell Biol. 89:792–802.

Lilley CE, Schwartz RA, Weitzman MD. 2007. Using or abusing: viruses and the cellular DNA damage response. Trends Microbiol. 15:119–126.

Luthra P, Ramanan P, Mire CE, Weisend C, Tsuda Y, Yen B, Liu G, Leung DW, Geisbert TW, Ebihara H, et al. 2013. Mutual antagonism between the ebola virus VP35 protein and the RIG-I activator PACT determines infection outcome. Cell Host Microbe 14:74–84.

Marzi A, Möller P, Hanna SL, Harrer T, Eisemann J, Steinkasserer A, Becker S, Baribaud F, Pöhlmann S. 2007. Analysis of the interaction of Ebola virus glycoprotein with DC-SIGN (dendritic cell-specific intercellular adhesion molecule 3-grabbing nonintegrin) and its homologue DC-SIGNR. J. Infect. Dis. 196 Suppl :S237–S246.

Maurer-Stroh S, Eisenhaber F. 2005. Refinement and prediction of protein prenylation motifs. Genome Biol. 6:R55.

McWilliam H, Li W, Uludag M, Squizzato S, Park YM, Buso N, Cowley AP, Lopez R. 2013. Analysis Tool Web Services from the EMBL-EBI. Nucleic Acids Res. 41.

Minguez P, Parca L, Diella F, Mende DR, Kumar R, Helmer-Citterich M, Gavin A-C, van Noort V, Bork P. 2012. Deciphering a global network of functionally associated post-translational modifications. Mol. Syst. Biol. 8:599.

Monigatti F, Gasteiger E, Bairoch A, Jung E. 2002. The Sulfinator: predicting tyrosine sulfation sites in protein sequences. Bioinformatics 18:769–770.

Mostafavi S, Ray D, Warde-Farley D, Grouios C, Morris Q. 2008. GeneMANIA: a real-time multiple association network integration algorithm for predicting gene function. Genome Biol. 9 Suppl 1:S4.

Munshi A, Ramesh R. 2013. Mitogen-Activated Protein Kinases and Their Role in Radiation Response. Genes Cancer 4:401–408.

Nishi H, Demir E, Panchenko AR. 2015. Crosstalk between signaling pathways provided by single and multiple protein phosphorylation sites. J Mol Biol 427:511–520.

Prins KC, Cárdenas WB, Basler CF. 2009. Ebola virus protein VP35 impairs the function of interferon regulatory factor-activating kinases IKKepsilon and TBK-1. J. Virol. 83:3069–3077.

Qiu W-R, Xiao X, Lin W-Z, Chou K-C. 2014. iMethyl-PseAAC: identification of protein methylation sites via a pseudo amino acid composition approach. Biomed Res. Int. 2014:947416.

Radivojac P, Vacic V, Haynes C, Cocklin RR, Mohan A, Heyen JW, Goebl MG, Iakoucheva LM. 2010. Identification, analysis, and prediction of protein ubiquitination sites. Proteins 78:365–380.

Saeed MF, Kolokoltsov A a, Freiberg AN, Holbrook MR, Davey R a. 2008. Phosphoinositide-3 kinase-Akt pathway controls cellular entry of Ebola virus. PLoS Pathog. 4:e1000141.

Seldin DC, Landesman-Bollag E, Farago M, Currier N, Lou D, Dominguez I. 2005. CK2 as a positive regulator of Wnt signalling and tumourigenesis. Mol Cell Biochem 274:63–67.

Shabman RS, Gulcicek EE, Stone KL, Basler CF. 2011. The Ebola virus VP24 protein prevents hnRNP C1/C2 binding to karyopherin ??1 and partially alters its nuclear import. J. Infect. Dis. 204.

Shabman RS, Leung DW, Johnson J, Glennon N, Gulcicek EE, Stone KL, Leung L, Hensley L, Amarasinghe GK, Basler CF. 2011. DRBP76 associates with Ebola virus VP35 and suppresses viral polymerase function. J. Infect. Dis. 204:911–918.

Shafman T, Khanna KK, Kedar P, Spring K, Kozlov S, Yen T, Hobson K, Gatei M, Zhang N, Watters D, et al. 1997. Interaction between ATM protein and c-Abl in response to DNA damage. Nature 387:520–523.

Sun L, Lv F, Guo X, Gao G. 2012. Glycogen synthase kinase 3β (GSK3β) modulates antiviral activity of zinc-finger antiviral protein (ZAP). J. Biol. Chem. 287:22882–22888.

Svegliati S, Marrone G, Pezone A, Spadoni T, Grieco A, Moroncini G, Grieco D, Vinciguerra M, Agnese S, Jüngel A, et al. 2014. Oxidative DNA damage induces the ATM-mediated transcriptional suppression of the Wnt inhibitor WIF-1 in systemic sclerosis and fibrosis. Sci. Signal. 7:ra84.

Tovchigrechko A, Vakser IA. 2006. GRAMM-X public web server for protein-protein docking. Nucleic Acids Res. 34.

Waterhouse AM, Procter JB, Martin DMA, Clamp M, Barton GJ. 2009. Jalview Version 2-A multiple sequence alignment editor and analysis workbench. Bioinformatics 25:1189–1191.

Wong YH, Lee TY, Liang HK, Huang CM, Wang TY, Yang YH, Chu CH, Huang H Da, Ko MT, Hwang JK. 2007. KinasePhos 2.0: A web server for identifying protein kinase-specific phosphorylation sites based on sequences and coupling patterns. Nucleic Acids Res. 35.

Xiaofei E, Kowalik TF. 2014. The DNA Damage Response Induced by Infection with Human Cytomegalovirus and Other Viruses. Viruses 6:2155–2185.

Xue Y, Chen H, Jin C, Sun Z, Yao X. 2006. NBA-Palm: prediction of palmitoylation site implemented in Naïve Bayes algorithm. BMC Bioinformatics 7:458.

Yang J, Yan R, Roy A, Xu D, Poisson J, Zhang Y. 2014. The I-TASSER Suite: protein structure and function prediction. Nat. Methods 12:7–8.

Zhang APP, Bornholdt Z a., Liu T, Abelson DM, Lee DE, Li S, Woods VL, Saphire EO. 2012. The ebola virus interferon antagonist VP24 directly binds STAT1 and has a novel, pyramidal fold. PLoS Pathog. 8.

Zhao Q, Xie Y, Zheng Y, Jiang S, Liu W, Mu W, Liu Z, Zhao Y, Xue Y, Ren J. 2014. GPS-SUMO: A tool for the prediction of sumoylation sites and SUMO-interaction motifs. Nucleic Acids Res. 42.
